# The acute vs. chronic effect of exercise on insulin sensitivity: nothing lasts forever

**DOI:** 10.1097/XCE.0000000000000239

**Published:** 2020-11-19

**Authors:** Fred J. DiMenna, Avigdor D. Arad

**Affiliations:** Division of Endocrinology, Diabetes and Bone, Department of Medicine, Icahn School of Medicine at Mount Sinai, New York, USA

**Keywords:** acute exercise effect, exercise, exercise is medicine, exercise prescription, exercise intensity, high-intensity interval training, insulin sensitivity, insulin resistance, prediabetes, type 2 diabetes

## Abstract

Regular exercise causes chronic adaptations in anatomy/physiology that provide first-line defense for disease prevention/treatment (‘exercise is medicine’). However, transient changes in function that occur following each exercise bout (acute effect) are also important to consider. For example, in contrast to chronic adaptations, the effect of exercise on insulin sensitivity is predominantly rooted in a prolonged acute effect (PAE) that can last up to 72 h. Untrained individuals and individuals with lower insulin sensitivity benefit more from this effect and even trained individuals with high insulin sensitivity restore most of a detraining-induced loss following one session of resumed training. Consequently, exercise to combat insulin resistance that begins the pathological journey to cardiometabolic diseases including type 2 diabetes (T2D) should be prescribed with precision to elicit a PAE on insulin sensitivity to serve as a first-line defense prior to pharmaceutical intervention or, when such intervention is necessary, a potential adjunct to it.

Video Abstract: http://links.lww.com/CAEN/A27

## Abstract 2

Supplemental Digital Content is available in the text.

## Introduction

The lingering effects of a single exercise bout (i.e. acute effect) can provide misleading information regarding a person’s health status in some circumstances. For example, if a habitually-sedentary person performed a bout of exercise the day before a physician’s visit, the prolonged acute effect (PAE) of exercise on insulin sensitivity could mask an elevated reading on an oral glucose tolerance test (OGTT). Conversely, for those who exercise regularly and, therefore, benefit from the ‘continuous’ presence of such an effect, the representation would be accurate. Importantly, this is also the case in the research setting where it is necessary to control for PAE when assessing previously-sedentary individuals; for example, before and after a training intervention if the objective is to determine the chronic effect that accumulates from repeat training (i.e. ‘training effect’). However, the need to discount the lingering effect of an exercise bout in these circumstances contrasts an acute effect’s potential role as a therapeutic agent for individuals who exercise frequently enough such that it is present most/all of the time. Moreover, in reality, the highly-touted chronic effect is also a bit of a misrepresentation because it, too, must be maintained. Granted, ‘detraining’ has a longer time course compared to dissipation of the acute effect; however, chronic adaptations do indeed wane if regular workouts are not continued. Finally, referring to either as a singular entity is an oversimplification because each likely includes multiple alterations in anatomy/physiology which might have different time courses for dissipation. For example, some adaptations comprising the acute effect might be longer lasting while some chronic changes are shorter lived. However, one thing is certain: when it comes to the effect of exercise on insulin sensitivity, nothing lasts forever.

## Type 2 diabetes and the obesity epidemic

Estimates indicate that ~34 million US citizens have diabetes [1] with 90–95% suffering from T2D [1]. Furthermore, it is estimated that ~88 million have prediabetes, which means blood-glucose concentration is higher than normal, but not yet high enough for T2D diagnosis (e.g. fasting level of 100–125 mg/dL compared to <100 and >125 for normal and T2D, respectively). Predictions suggest up to 70% of individuals with prediabetes will develop T2D [2] such that total prevalence in 2050 could increase to as much as 33% of the population (compared to, for example, 14% in 2010) [3]. Intervention at the prediabetic stage is, therefore, critical. Excess body-fat deposition is a risk factor for T2D with 10-year risk increasing by 4.6- and 10.0-fold for females and 3.5- and 11.2-fold for males classified as overweight (BMI, 25.0–29.9 kg/m^2^) or obese (BMI, 30.0–34.9 kg/m^2^), respectively [4]. However, ‘metabolically-healthy obese’ and ‘metabolically-obese normal-weight’ (MONW) phenotypes indicate greater complexity [4] at least partially rooted in genetic predisposition [5].

## Exercise is medicine

‘Exercise is medicine’ is an initiative launched by the American Medical Association and American College of Sports Medicine in 2007 [6]. The objective was to make physical-activity assessment/promotion standard in clinical practice so that healthcare can be augmented by evidence-based physical-activity resources [6]. Given the prevalence of insulin resistance and its association with obesity as a disorder of fuel storage/usage, exercise is medicine might be particularly appropriate for addressing the T2D epidemic [7]. Insulin-stimulated glucose uptake is primarily directed to skeletal muscle [8] and, in addition to energy use during exercise, post-exercise recovery and the additional fat-free mass gained by sedentary individuals who begin exercising each serve to favorably alter energy balance. This could prove beneficial for prevention/treatment of these diseases. However, to isolate the influence of chronic exercise per se on insulin sensitivity, in addition to differentiating chronic from acute effect, it is important to consider the independent effect of body-fat loss that often occurs when sedentary individuals begin exercising. When body fat is reduced, insulin resistance improves even when reduction occurs via surgical intervention [9,10]. Conversely, some evidence suggests that chronic exercise neither augments the improvement in insulin sensitivity induced by weight loss due to dietary restriction without exercise [11,12; *c.f.* 13] nor improves insulin sensitivity when weight loss is prevented [14–17; *c.f.* 18]. This suggests that the chronic effect of exercise per se might be insufficient as medicine for insulin resistance. Furthermore, considering the growing appreciation for location in addition to quantity of fat storage [19,20], the ability for chronic exercise to redistribute fat is also important to consider. Finally, some of the structural changes that result in improved insulin sensitivity consequent to chronic training might be unattainable for individuals most in need of an intervention to combat insulin resistance. Collectively, these complexities associated with the chronic effect coupled with the relatively ‘simple’ (not mechanistically but from a practical standpoint) influence an individual session can evoke implies that the acute effect might be more relevant to consider when prescribing exercise to combat insulin resistance.

## Contraction-mediated insulin-independent glucose uptake

The acute effect of exercise as a stimulant for glucose uptake by skeletal muscle begins during exercise when a number of factors work in concert to facilitate an increase [21]. Exercise-induced increased perfusion to and capillary recruitment within contracting muscle allows for greater glucose delivery/exchange [22] while contraction-mediated insulin-independent activation of intracellular signaling pathways results in glucose extraction which can exceed that which is present with maximal insulin stimulation depending on muscle fibre type [22]. Glucose transport occurs via facilitated diffusion utilising glucose transporter isoform 4 (GLUT-4), a protein found in muscle and adipose tissue [23]. Similar to insulin stimulation, contractions increase GLUT-4 translocation to the sarcolemma of muscle fibers; however, in this case, there is evidence which indicates that GLUT-4 might emanate from a different intracellular pool [24]. This distinction is important because it suggests that the effects of insulin and contractions are additive [25]. Exercise-induced activation of GLUT-4 is predominantly driven by AMP-activated protein kinase (AMPK; an enzyme that plays an important role in regulating cellular energy status) [26] and Rac1, a member of the guanosine triphosphatase (GTP) family of regulatory proteins [27]. However, other pathways (e.g. related to calcium, reactive oxygen species and nitric oxide signaling) appear to provide protective redundancy [28]. During exercise, increased AMPK results in deactivation of tre-2/USP6, BUB2, cdc16 domain family member 1 (TBC1D1), a member of the Rab GTPase-activating protein family [29]. Deactivation of TBC1D1 removes its inhibitory influence thereby promoting GLUT-4 translocation [29,30]. This ‘early phase’ comprising insulin-independent glucose uptake during exercise persists after the bout for 30 (but not 120) min [31]; however, the acute effect of exercise on insulin sensitivity also includes long-lasting enhancement that can persist for up to 72 h [32]. As previously mentioned, this PAE of exercise on insulin sensitivity might be most relevant when prescribing exercise as medicine for insulin resistance.

### The prolonged acute exercise effect on insulin sensitivity: mechanistic bases

Four hours following exercise, insulin-stimulated GLUT4 translocation and glucose uptake by rat skeletal muscle is increased compared to the pre-exercise condition in association with increased phosphorylation of Akt substrate of 160 kDa (AS160; also known as TBC1D4), a Rab GTPase-activating protein paralogue [33] that appears to be ‘primed’ by AMPK [30]. However, unlike the insulin-independent route, TBC1D1 is not directly involved in this process [33]. For example, Kjøbsted *et al*. recently confirmed that 3 h after contraction or 6 h after stimulation of 5-Aminoimidazole-4-carboxamide ribonucleotide (the first direct AMPK activator), the increase in insulin-stimulated glucose uptake driven by enhanced insulin sensitivity in muscle of wild-type mice is absent in mice lacking TBC1D4 even though the phosphorylation pattern of TBC1D1 in these animals was unaltered [30]. Moreover, this prolonged post-exercise effect on insulin sensitivity occurs with unaltered insulin binding or insulin receptor substrate (IRS) activation (i.e. proximal insulin signaling) [30]; however, a simple cause-effect relationship between events at/distal to AS160 and the exercise-induced increase in insulin-stimulated glucose uptake has not been clarified and likely involves greater complexity [29]. To differentiate the PAE of exercise on insulin sensitivity from the chronic effect, Prior *et al*. had previously-sedentary older individuals perform aerobic training for 6 months followed by 2 weeks of detraining to ‘wash out’ PAE and found that insulin sensitivity (as evaluated during euglycemic hyperinsulinemic clamp; EHC) increased by ~25% after training with an ~18% increase still present after detraining [34]. Reduction of the training-induced increase from ~25 to ~18% after 2 weeks of detraining indicates that ~25% of the improvement that was achieved due to 6 months of regular training was attributable to short-term effects [34]. Mechanistically, the loss of these short-term effects occurred in conjunction with return of GLUT-4 expression, AMPKα1 expression and insulin-mediated activation of glycogen synthase to baseline levels whereas the increase that remained was associated with an increase in capillary density that training elicited [34]. Importance of microvascular structural changes as a chronic adaptation to exercise training for improved glycemic control agreed with earlier observations by Williamson *et al*. who found that the increase in width of the capillary basement membrane that occurs with sedentary aging is normalised after 9 months of endurance training regardless of baseline glucose tolerance [35]. However, being that body-fat modulates insulin resistance, especially when it is located in the abdominal region [36], the most important factor that positively influences insulin sensitivity as a chronic adaptation to exercise training appears to be the change in body composition that typically accompanies it [11,12,14–17].

### The effect of exercise on insulin sensitivity: early findings

In a landmark study, Heath *et al*. found that when chronically-trained individuals with high insulin sensitivity performed no exercise for 10 days, an OGTT revealed a reduction in insulin sensitivity compared to the trained state despite no change in fitness level (as indicated by maximal rate of oxygen uptake; ${\dot{\text{V}}\text{o}}_{2\max})$ or body weight/fat [37]. However, a single bout following detraining resulted in an insulin response that was not different at any post-exercise time point (30, 60, 120 and 180 min) compared to the trained state [37]. Collectively, the marked reduction in insulin sensitivity following a short period of detraining and almost complete restoration of the ‘trained insulin sensitivity’ after the initial bout of resumption indicates the important role that the PAE of exercise plays in maintaining insulin sensitivity even for trained individuals [37].

The relevance of the PAE in relation to the overall influence of exercise on insulin sensitivity reported by Heath *et al*. provided information in humans that complemented early research in animals. For example, 1 year prior, Richter *et al*. observed greater glucose utilisation in the isolated perfused hindlimb muscle for 4 h following 45 min of treadmill running by untrained rats when insulin was added to the perfusate [38]. Importantly, glucose utilisation was unchanged compared to the no-exercise control condition in the absence of added insulin which confirmed that acute exercise influenced the insulin-dependent pathway [38]. In a follow-up study, Ivy *et al*. compared glucose uptake at various insulin concentrations in the isolated perfused hindlimb muscle of rats that were trained for 14–16 weeks and found that the rate of glucose uptake the day after the final training session was ~50% greater than that which was present in sedentary controls [39]. However, 40–46 h later, there was no difference regardless of the presence of insulin at physiological or maximally-effective levels [39]. Furthermore, a swimming bout to exhaustion and muscle contractions induced by electrical stimulation both resulted in an increase in glucose uptake the following day that was of similar magnitude regardless of whether the rat was trained or untrained [39]. The authors concluded that the increase in insulin-stimulated glucose uptake demonstrated by chronically-trained rats is a function of the PAE of exercise on insulin sensitivity as opposed to a long-term adaptive response [39]. In 1989, Cartee *et al*. provided further insight when they found that the PAE of exercise on insulin sensitivity could persist for up to 48 h in rats subject to carbohydrate deprivation whereas high-carbohydrate feeding during the post-exercise period resulted in more rapid dissipation [40]. This was consistent with earlier findings which indicated that insulin-stimulated carbohydrate storage rate is increased after glycogen-depleting exercise without, but not with re-feeding, even though post-exercise carbohydrate oxidation rate is decreased in both cases [41].

More information regarding the PAE of exercise on insulin sensitivity in humans was provided by Rogers *et al*. in 1990 [42]. These researchers replicated the 10-day detraining methodology employed by Heath *et al*. (see above); however, in this case, their subjects were 14 masters athletes whose life-long training allowed them to avoid the development of insulin resistance that typically accompanies aging. Interestingly, for 10 of these athletes, responses for glucose and insulin after 10 days of detraining were not different than those of young, lean controls [42]. This meant that for these individuals, in addition to the PAE of exercise on insulin sensitivity, exercise training conveyed a chronic effect that protected them from aging-related insulin resistance. The authors speculated that the positive effect of chronic exercise on body fatness was responsible [42]. However, despite similar leanness, the other four athletes experienced a reduction in glucose tolerance due to detraining which confirmed that for these individuals, the PAE of exercise was responsible for their ‘youthful’ insulin sensitivity [42]. The authors speculated that this subgroup had a genetic predisposition to develop insulin resistance that was offset by the PAE of exercise [42]. This supports the contention that for individuals most at need for an intervention to maintain insulin sensitivity/prevent insulin resistance, it is the PAE as opposed to chronic effect of exercise on insulin sensitivity that is the more relevant to consider. However, in a previous study, these same researchers found that the improvement in OGTT demonstrated by individuals with mild T2D 18 h after the final bout of a 7-day aerobic training intervention was not yet present following the initial session [43]. This suggests greater complexity that might be rooted in the counterbalancing effect of reduced insulin concentration or the intensity of the exercise being performed [43]. Nevertheless, the fact that insulin sensitivity was improved after 7 days (i.e. a training period that was too short to result in changes in body composition or ${\dot{\text{V}}\text{o}}_{2\max})$ indicates that the PAE of exercise on insulin sensitivity (albeit apparently derived from the accumulation of multiple bouts) was capable of providing therapeutic benefit for patients with T2D [43].

Definitive proof that the improved insulin action demonstrated by endurance-trained individuals was due to increased insulin sensitivity was provided by King *et al*. in 1987 [44]. These researchers used a two-stage (i.e. submaximal and maximal stimulation) EHC to tease out the difference between changes in insulin sensitivity per se as opposed to changes in responsiveness to maximal insulin stimulation [44]. In a follow-up study, the same group also confirmed that the superior insulin sensitivity demonstrated by trained individuals is predominantly transient as the same rate of whole-body glucose disposal required an ~67% higher plasma insulin response after 14 days of inactivity compared to 16 h post-exercise [45]. Finally, in addition to these longitudinal studies, a cross-sectional analysis by Helmrich *et al*. confirmed that leisure-time physical activity (quantified as energy expended per week in walking, stair climbing, and sports) was inversely related to the development of T2D for 5990 male alumni of the University of Pennsylvania during 98 524 man-years of follow-up from 1962 to 1976 [46]. Importantly, this protective effect of physical activity was strongest in persons with high BMI, a history of hypertension or a parental history of diabetes, which confirmed that those at greatest risk for developing T2D stood to benefit most from exercise training per se as a preventive intervention [46].

## The prolonged acute exercise effect on insulin sensitivity: recent findings

Recent research provides more insight regarding the effect of exercise on insulin sensitivity and, specifically, the importance of the PAE in this regard. In 2019, Steenberg *et al*. had healthy men perform one-legged knee-extensor exercise at the same relative intensity before and after 12 weeks of bilateral cycle training and observed enhanced insulin sensitivity (EHC) 4–6 h after exercise in the trained compared to untrained state [47]. However, in the trained state, when insulin sensitivity was compared between tested (PAE plus chronic effect) and untested (chronic effect only) leg, the difference between the two was reduced by ~50% compared to the difference that was present pre-training (i.e. when tested and untested leg were experiencing only the PAE and no effect, respectively) [47]. This implies that the chronic effect occurred at the ‘expense’ of the PAE that was present in the untrained state [47]. One possible explanation is that in the trained state, exercise-induced AMPK-mediated phosphorylation of TBC1D4 was decreased despite the fact that pre- and post-training bouts were performed at the same ‘relative’ intensity [47]. Regardless of mechanistic basis; however, assuming that the exercised muscle’s rate of glucose uptake in the trained state was not maximal (i.e. there was ‘room’ for a further increase), reciprocity between acute and chronic enhancements implies that the positive influence of exercise per se on insulin sensitivity is constrained by an upper limit that cannot be surpassed [47]. A similar conclusion can be drawn from the work of Nelson and Horowitz who found that the superior insulin sensitivity (i.e. compared to sedentary individuals with similar body composition; however, still inferior compared to healthy lean individuals) displayed by chronically-trained overweight/obese individuals after 3 days of detraining (i.e. when only the chronic effect should still be present) was not further enhanced by the PAE following one bout of resumption of training whereas the PAE following the single bout increased insulin sensitivity to the same level for their sedentary counterparts [48]. Collectively, these findings support the contention that while chronic effects from accumulated training should not be discounted, apparently, as Cartee asserts, ‘once is enough’ to benefit insulin sensitivity [49], particularly for individuals most in need of a preventive/treatment intervention.

## Eliciting the prolonged acute exercise effect on insulin sensitivity

‘Physical activity’ refers to bodily movement by skeletal muscle which results in energy expenditure [50]. ‘Exercise’ has been defined as a subset of physical activity because it must be planned, structured and repetitive, and have a final or intermediate objective of improving or maintaining physical fitness [50]. While more specific, this classification is still broad and the term, therefore, describes many types of physical activities that individuals perform. While such variety likely improves program adherence [51], if exercise is to represent a true alternative to pharmaceutical intervention [6], it must be ‘prescribed’ with greater rigor [7]. For example, the objective in the research setting should be to define exercise for eliciting the PAE of exercise on insulin sensitivity with evidence-based precision by better identifying the most effective mode (e.g. endurance or resistance training and in the case of the former, lower-body exclusive like the oft-performed leg cycling or full body), duration (the time for which exercise must be maintained during each individual session; e.g. a minimal and optimal dose), frequency (based on how long the effect lasts, elucidation of which requires investigations with measurements of insulin sensitivity at multiple time points post-exercise) and intensity (e.g. low sustained for long duration, moderate/high sustainable for lesser duration or very high unsustainable intermittent) with participant characteristics and post-exercise feeding taken into account. Table 1 provides a chronologically-ordered list of studies that have contributed to our current understanding of the PAE of exercise on insulin sensitivity which is important to consider when making these determinations and planning future research.

**Table 1:** A chronologically-ordered listing of studies that have contributed to our current understanding of the acute effect of exercise on insulin sensitivity in different human populations

Study	Participants	Exercise			Insulin sensitivity assessment		Exercise effect
		Mode	Volume	Intensity	Time post-exercise	Method	
Devlin and Horton (1985) [52]	Obese IGT or hyperinsulinemia untrained males (*n* = 6) vs. lean healthy untrained males (*n* = 6)	Cycle	2 min on/3 min off to limit of tolerance (obese, 33 ± 2 min; lean, 66 ± 3 min)	85% ${\dot{\text{V}}\text{o}}_{2\max}$	12–16 h	EHC	↑ Insulin sensitivity post bout for obese, but not for lean
Devlin *et al*. (1987) [53]	T2D untrained males (*n* = 5)	Cycle	2 min on/3 min off to limit of tolerance (41 ± 10 min)	85% ${\dot{\text{V}}\text{o}}_{2\max}$	12–16 h	EHC	↑ Insulin sensitivity post bout
Mikines *et al*. (1988) [54]	Healthy untrained young males (*n* = 7)	Cycle	60 min	150 W	0 h, 48 h, 120 h	EHC	↑ Insulin sensitivity at 48 h post bout; ↔ insulin sensitivity at 120 h post bout
Richter *et al*. (1989) [55]	Healthy young RA males (*n* = 6)	One-legged knee-extensor exercise	60 min	75% W_max_	4 h	EHC	↑ Insulin sensitivity post bout for exercised, but not for rested leg
Young *et al*. (1989) [56]	Healthy trained males post ≥40 h detraining (*n* = 7) vs. healthy RA males (*n* = 7)	Cycle	40 min	~40% ${\dot{\text{V}}\text{o}}_{2\max}$	14 h	OGTT	↓ I-AUC/↔ G-AUC post bout for untrained so that they were no longer different vs. trained who displayed superior I-AUC pre bout that was unchanged post bout; no difference between conditions
		Cycle	40 min	~80% ${\dot{\text{V}}\text{o}}_{2\max}$		(I-AUC/G-AUC)	
Burnstein *et al*. (1990) [57]	Obese T2D non-AA males (*n* = 3) and females (*n* = 3) vs. obese NG non-AA males (*n* = 2) and females (*n* = 5) vs. lean healthy non-AA males (*n* = 6)	Treadmill	60 min	HR = 150–160 bpm	60 min	EHC	↑ Insulin sensitivity post bout for obese T2D and NG; ↔ insulin sensitivity post bout for lean
Kjaer *et al*. (1990) [58]	Healthy sedentary males (*n* = 7) vs. T2D sedentary males (*n* = 7)	Cycle	7 min + 3 min + 2 min	~60% ${\dot{\text{V}}\text{o}}_{2\max}$	24 h	EHC	↑ Insulin sensitivity post bout for T2D, but not for healthy
				~100% ${\dot{\text{V}}\text{o}}_{2\max}$			
				~110% ${\dot{\text{V}}\text{o}}_{2\max}$			
Fluckey *et al*. (1994) [59]	Young healthy untrained males (*n* = 3) and females (*n* = 4) vs. older T2D untrained males (*n* = 3) and females (*n* = 4)	Machine full-body resistance exercise	3 sets/exercise × 10 reps or to failure if it occurred sooner	50, 75 and 100% of calculated	18 h	OGTT(I-AUC/G-AUC)	↓ I-AUC/↔ G-AUC post bout for both groups
				10-RM			
Ben Ezra *et al*. (1995) [60]	Healthy untrained females (*n* = 24)	Treadmill	~87 min	~40% ${\dot{\text{V}}\text{o}}_{2\max}$	15–17 h	OGTT	↔ I-AUC/↔ G-AUC post bout for MIE; ↓ I-AUC/↔ G-AUC post bout for HIE
			~50 min	~70% ${\dot{\text{V}}\text{o}}_{2\max}$		(I-AUC/G-AUC)	
Braun *et al*. (1995) [61]	Obese T2D untrained females (*n* = 8)	Treadmill	143 ± 23 min (3 bouts over 2 days)	50 ± 2% ${\dot{\text{V}}\text{o}}_{2\max}$	~18 h	IST	↑ Insulin sensitivity post bout for both conditions; no difference between conditions
			90 ± 8 min (3 bouts over 2 days)	74 ± 3% ${\dot{\text{V}}\text{o}}_{2\max}$			
Perseghin *et al*. (1996) [62]	Young lean non-HPA non-excessively sedentary male (*n* = 6) and female (*n* = 2) offspring of T2D vs. young lean non-HPA non-excessively sedentary males (*n* = 3) and females (*n* = 5) with no T2D family history	Stair climbing	3 × 15 min	~65% ${\dot{\text{V}}\text{o}}_{2\max}$	48 h	HHC	↑ Insulin sensitivity post bout for both groups
Wojtaszewski *et al*. (2000) [63]	Healthy young males (*n* = 7)	One-legged knee-extensor exercise	60 min	75–95% W_max_	4 h	EHC	↑ Insulin sensitivity post bout for exercised, but not rested leg
Chapman *et al*. (2002) [64]	Sedentary PM females (*n* = 10)	Machine full-body resistance exercise	3 sets × 10 reps or failure if sooner	50, 75 and 100% calculated 10-RM	14 h	IVGTT (minimal model)	↔ Insulin sensitivity post bout
Fenicchia *et al*. (2004) [65]	Healthy untrained females (*n* = 8) vs. T2D untrained females (*n* = 7)	Free weight and machine full-body resistance exercise	3 sets × 8–12 reps to failure	80% 3-RM	12–24 h	OGTT	↔ I-AUC/↓ G-AUC post bout for T2D; ↔ I-AUC/↔ G-AUC post bout for healthy
						(I-AUC/G-AUC)	
Koopman *et al*. (2005) [66]	Healthy untrained males (*n* = 12)	Machine leg resistance exercise	8 sets × 10 reps	75%	24 h	ITT	↑ Insulin sensitivity post bout
				1-RM		(ΔBG_16-4_)	
						and	
						HOMA	
O’Gorman *et al*. (2006) [67]	Obese T2D males (*n* = 6) and females (*n* = 2) vs. obese NGT males (*n* = 6) and females (*n* = 1)	Cycle	60 min	~75% ${\dot{\text{V}}\text{o}}_{2\text{peak}}$	16 h	EHC	↔ Insulin sensitivity post single bout (acute effect); ↑ insulin sensitivity post final bout of 7 performed over 7 days (chronic effect)
Zhang *et al*. (2006) [68]	MS sedentary males (*n* = 10)	Treadmill	60 min	~40% ${\dot{\text{V}}\text{o}}_{2\max}$	14–20 h	Post fat-meal	↔ Insulin sensitivity post bout/fat meal for MIE; ↑ insulin sensitivity post bout/fat meal for both HIE
			60 min	~60% ${\dot{\text{V}}\text{o}}_{2\max}$		HOMA	
			60 min	~70% ${\dot{\text{V}}\text{o}}_{2\max}$			
Howlett *et al*. (2007) [69]	RA males (*n* = 8)	Machine leg resistance exercise	3 sets × 10–12 reps	80% 1-RM	0 h	EHC	↓ Insulin sensitivity post single bout (acute effect); ↔ insulin sensitivity post final bout of 4 performed over 7 days (chronic effect)
Schenk and Horowitz (2007) [70]	Healthy inactive females (*n* = 8)	Treadmill +	45 min +	66 ± 1% ${\dot{\text{V}}\text{o}}_{2\text{peak}}$	15–18 h	IVGTT (minimal model)	↑ Insulin sensitivity (i.e. removal of lipid-induced impairment + enhancement vs. baseline) in response to overnight lipid infusion post bout
		cycle	45 min				
Magkos *et al*. (2008) [71]	Healthy RA males (*n* = 30)	Cycle or treadmill	30, 60, 90 or 120 min	61–63 ± 2% ${\dot{\text{V}}\text{o}}_{2\text{peak}}$	12 h	HOMA	↑ Insulin sensitivity post bout for ≥900 kcal expenditure; ↔ insulin sensitivity post bout for <900 kcal expenditure
Brestoff *et al*. (2009) [72]	Healthy RA young males (*n* = 8) and females (*n* = 5)	Cycle	3 × 15 min	~75% ${\dot{\text{V}}\text{o}}_{2\text{peak}}$	12–16 h	OGTT	↑ Insulin sensitivity post bout for 75% ${\dot{\text{V}}\text{o}}_{2\text{peak}}$_;_ ↔ insulin sensitivity post all-out
		Cycle	5 × 30 s	All-out		(Matsuda) and HOMA	
Black *et al*. (2010) [73]	IFG sedentary males (*n* = 12) and females (*n* = 5)	Free weight and machine full-body resistance exercise	1 set or 4 sets × 12–15 reps/exercise	65% 1-RM	24 h	HOMA	↑ Insulin sensitivity post bout for all protocols; 85% 1-RM > 65% 1-RM and 4 sets > 1 set
			1 set or 4 sets × 6–8 reps/exercise	85% 1-RM			
Hasson *et al*. (2010) [74]	Healthy sedentary black males (*n* = 4) and females (*n* = 7) vs. healthy sedentary white males (*n* = 4) and females (*n* = 6)	Treadmill	4 × 15 min	75% HR_max_	12–15 h	EHC	↔ Insulin sensitivity post bout for both groups
Manders *et al*. (2010) [75]	T2D sedentary males (*n* = 10)	Cycle	60 min	35% W_max_	24 h	OGTT	↓ 24-h average glucose concentration post 35% W_max;_ ↔ 24-h average glucose concentration post 70% W_max_
						(OGIS)	
		Cycle	30 min	70% W_max_		and	
						HOMA	
Newsom *et al*. (2010) [76]	Healthy sedentary males (*n* = 9)	Treadmill +	45 min +	60–65% ${\dot{\text{V}}\text{o}}_{2\text{peak}}$	24 h	IVGTT (minimal model)	↔ Insulin sensitivity post bout when energy and carbohydrate balance were maintained and when carbohydrate balance was maintained with energy deficit; ↑ insulin sensitivity post bout when energy balance was maintained with carbohydrate deficit
		Cycle	45 min				
Richards *et al*. (2010) [77]	Healthy sedentary/RA young males (*n* = 2) and females (*n* = 7)	Cycle	4 × 30 s	All-out	72 h	EHC	↔ Insulin sensitivity post single bout (acute effect); ↑ insulin sensitivity post final bout of 6 performed over 14 days (chronic effect)
Short *et al*. (2012) [78]	Healthy young RA/non-RA (*n* = 4/7) males (*n* = 4) and females (*n* = 7)	Treadmill +	15 min +	75% HR_max_	1 h, 17 h	IVGTT (Minimal Model) and MMTT (Matsuda)	↑ Insulin sensitivity in response to meal post bout at 1 h, but not 17 h
		cycle + video-game boxing	15 min + 15 min				
Newsom *et al*. (2013) [79]	Obese sedentary males (*n* = 3) and females (*n* = 8)	Treadmill + cycle	70 ± 3 min	51% ${\dot{\text{V}}\text{o}}_{2\text{peak}}$	19 h	EHC	↑ Insulin sensitivity post bout for MIE; ↔ insulin sensitivity post bout for HIE; no difference between conditions; Δ insulin sensitivity correlated with Δ fatty-acid disappearance from plasma
			54 ± 2 min	66% ${\dot{\text{V}}\text{o}}_{2\text{peak}}$			
Whyte *et al*. (2013) [80]	Overweight/obese sedentary males (*n* = 10)	Cycle	4 × 30 s	All out	18–22 h	OGTT (Matsuda) and HOMA	↑ Insulin sensitivity post bout when work was performed in one continuous bout; ↔ insulin sensitivity post bout when work was performed in 4 bouts separated by 270 s
			1 × 198 ± 10 s	All out			
De Matos *et al*. (2014) [81]	Lean healthy untrained males (*n* = 3) and females (*n* = 6) vs. obese-insulin sensitive healthy untrained males (*n* = 3) and females (*n* = 6) vs. obese-insulin resistant untrained males (*n* = 3) and females (*n* = 6)	Cycle	3 × 20 min	50% W_peak_	30 min	Biopsy (western blot for measurement of protein expression of cellular stressors related to insulin resistance; no direct measurement of insulin sensitivity)	↓ JNK post bout for obese-insulin resistant; ↔ JNK post bout for healthy lean and obese-insulin sensitive; ↓ p-IRS-1^ser612^ post-bout for obese-insulin resistant and obese-insulin sensitive; ↔ p-IRS-1^ser612^ for healthy lean; ↑HSP70 post-bout main effect
Nelson and Horowitz (2014) [48]	Overweight/obese male (*n* = 5) and female (*n* = 7) exercisers post 72 h detraining vs. overweight/obese male (*n* = 5) and female (*n* = 7) non-exercisers	Treadmill	60 min	70% APMHR	24 h	OGTT (Matsuda)	↑ Insulin sensitivity post bout for non-exercisers so that they were no longer different vs. exercisers who displayed superior insulin sensitivity pre-bout that was unchanged post bout
Rynders *et al*. (2014) [82]	Pre-T2D sedentary males (*n* = 9) and females (*n* = 9)	Cycle	40 ± 9 min	~50%${\dot{\text{V}}\text{o}}_{2\text{peak}}$~83% ${\dot{\text{V}}\text{o}}_{2\text{peak}}$	60 min	OGTT (Minimal Model)	↑ Insulin sensitivity post bout that was not different between conditions
			24 ± 5 min				
Ortega *et al*. (2015) [83]	Healthy, young RA males (*n* = 10)	Cycle	4 × 30 s	All out	30 min, 24 h, 48 h	IVGTT	↑ Insulin sensitivity 30 min, 24 h and 48 h post bout for all conditions; all out ↑ > continuous ↑ at 30 min
			60 min	46 ± 3% ${\dot{\text{V}}\text{o}}_{2\text{peak}}$			
			35 ± 2 min	77 ± 5% ${\dot{\text{V}}\text{o}}_{2\text{peak}}$			
Malin *et al*. (2016) [84]	Pre-T2D middle-aged sedentary males (*n* = 7) and females (*n* = 8)	Treadmill	42 ± 2 min	68 ± 1% ${\dot{\text{V}}\text{o}}_{2\text{peak}}$	60 min	OGTT (Minimal Model)	↓ Insulin resistance-SM post bout that was not different between conditions; ↔ insulin resistance-HEP and insulin resistance-AT post bout for MIE; ↑ insulin resistance-HEP and insulin resistance-AT post bout for HIE
			24 ± 1 min	90 ± 1% ${\dot{\text{V}}\text{o}}_{2\text{peak}}$			
Metcalfe *et al*. (2016) [85]	Healthy sedentary/RA young males (*n* = 8) and females (*n* = 6)	Cycle	3 × 15 min	76 ± 4% ${\dot{\text{V}}\text{o}}_{2\max}$	14–16 h	OGTT (Cederholm)	↔ Insulin sensitivity post bout for both conditions
			5 × 30 s	All out			
Castleberry *et al*. (2019) [86]	Healthy inactive males (*n* = 10)	Treadmill	1 × 60 min	78 ± 6% ${\dot{\text{V}}\text{o}}_{2\max}$	12–14 h	OGTT (Matsuda)	↔ Insulin sensitivity post bout(s) for all conditions
			3 × 60 min (consecutive days)	78 ± 6% ${\dot{\text{V}}\text{o}}_{2\max}$			
			3 × 60 min (alternate days)	77 ± 5% ${\dot{\text{V}}\text{o}}_{2\max}$			

AA, athletically active; APMHR, age-predicted maximum heart rate; bpm, beats per minute; Cederholm, Cederholm index; Δ, change in variable, ΔBG_16-4_, change in blood-glucose concentration from 4 to 16 min; EHC, euglycemic hyperinsulinemic clamp; G-AUC, glucose area under the curve; HHC, hyperglycemic hyperinsulinemic clamp; HIE, high-intensity exercise; HOMA, homeostatic model assessment; HPA, participates in heavy physical activity; HR, heart rate; HR_max_, maximum heart rate; HSP70, heat shock protein 70; I-AUC, insulin area under the curve; IFG, impaired fasting glucose; IGT, impaired glucose tolerance; Insulin resistance-AT, adipose tissue insulin resistance; Insulin resistance-HEP, hepatic insulin resistance; Insulin resistance-SM, skeletal muscle Insulin resistance; IST, insulin suppression test; ITT, insulin tolerance test; IVGTT, intravenous glucose tolerance test; JNK, C-jun N-terminal kinase phosphorylation; Matsuda, Matsuda index; MIE, moderate-intensity exercise; MMTT, mixed meal tolerance test; MS, with metabolic syndrome; NG, normoglycemic; NGT, normal glucose tolerance; OGIS, oral glucose insulin sensitivity index; OGTT, oral glucose tolerance test; p-IRS-1^ser612^, insulin receptor substrate 1 serine 612 phosphorylation; PM, postmenopausal; Pre-T2D, with prediabetes; RA, recreationally active; RM, repetition maximum; T2D, with type 2 diabetes mellitus; ${\dot{\text{V}}\text{o}}_{2\text{peak}}$, peak rate of oxygen uptake; ${\dot{\text{V}}\text{o}}_{2\max}$, maximum rate of oxygen uptake; W_max_, maximum work rate.

## Mode

Studies investigating the PAE of exercise on insulin sensitivity have typically involved endurance (‘aerobic’) exercise; specifically, leg-cycle ergometry, treadmill exercise or a combination of the two (see Table 1). This stands to reason being that cycling and walking/jogging are the most accessible modalities for the average person. Furthermore, exercise’s acute insulin-sensitising effect is mediated by local (as opposed to systemic) contraction-induced factors [87,88]; hence, modalities like these which require sustained involvement of a greater quantity of muscle and larger muscles would likely be superior. Resistance (‘strength’) training can involve larger muscles; however, it can also be performed for upper-body musculature resulting in activation of more total muscle mass. Given that resistance training induces muscular hypertrophy and skeletal muscle is the predominant site for glucose disposal [89], it is not surprising that chronic resistance training improves insulin sensitivity [90]. However, unlike endurance exercise, the acute (and, therefore, not hypertrophy-/body-composition related) effect of resistance training has not been investigated frequently and findings are equivocal. With respect to the ability to improve glycemic control, van Dijk *et al*. used subcutaneous glucose monitoring following a single 45-min bout of endurance (cycling at 50% maximum work rate) or resistance (75% one-repetition maximum) exercise performed by overweight/obese males with impaired glucose tolerance or treated T2D and found that hyperglycemic glucose concentrations were similarly lowered in all three groups for 24 h after both types of exercise [91]. As for insulin action, Fluckey *et al*. found that unlike older healthy individuals, older individuals with T2D and young control participants each demonstrated increased insulin clearance as evidenced by a reduced total insulin response (area under curve; AUC) with no change in C-peptide response (an indirect index of insulin secretion) during OGTT 18 h after resistance training [59]. Fenicchia *et al*. assessed women with T2D and healthy controls and found that for the former, but not the latter, full-body resistance training reduced glucose AUC and peak glucose concentration (OGTT) 12–24 h post-exercise with no change in insulin concentration [65]. Finally, Black *et al*. found that individuals with impaired fasting glucose demonstrated increased insulin sensitivity (homeostatic model assessment; HOMA) 24 h following two different full-body resistance training bouts [73]. Conversely, Chapman *et al*. observed no change compared to the no-exercise control condition for insulin sensitivity or AUC for glucose or insulin during an intravenous glucose tolerance test (IVGTT) for sedentary postmenopausal women with normal glucose tolerance 15 h after resistance training [64]. In addition to metabolic health, these discrepant findings might be attributable to participant age, gender [64] or the method used to assess insulin sensitivity [66]. Specifically, with respect to the latter, instead of OGTT or IVGTT, Koopman *et al*. used an intravenous insulin tolerance test (ITT) and found that healthy male participants demonstrated increased insulin sensitivity 24 h post-resistance training that mainly consisted of leg exercises [66]. In addition to testing method, resistance training intensity and control of pre-test diet and physical activity levels might be responsible for this finding [67]. However, large intersubject variability and absence of response in some participants suggest greater complexity [67]. Finally, Howlett *et al*. found that compared to the no-exercise condition, insulin sensitivity (EHC) was unchanged 24 h following the final of three resistance training bouts performed over the course of a week by young recreationally-active men [69]. Interestingly, the authors also observed decreased insulin sensitivity immediately following the initial session (i.e. reversal of the immediate acute-exercise effect after endurance exercise; see above) in association with a decrease in AS160 phosphorylation [69]. These findings and others [92] indicate that even if resistance training provides a viable alternative to endurance exercise for inducing the PAE of exercise on insulin sensitivity, the post-exercise response by signaling pathways that affect glucose metabolism is different. In this regard, using an electrical stimulation resistance training model in rats, Kido *et al*. recently found that the increase in insulin-stimulated glucose uptake 6 h after a resistance-training bout is greater when mechanistic target of rapamycin complex 1 (mTORC1) and associated downstream phosphorylation of IRS-1 serine residues (IRS-1 Ser) is inhibited [93]. This has resonance because IRS-1 Ser phosphorylation is associated with insulin resistance. Importantly, compared to endurance exercise, mTORC1 activation, which stimulates skeletal-muscle hypertrophy, is higher and has a longer duration after resistance training. Collectively, this implies that the mTORC-1-induced phosphorylation of IRS-1 Ser and consequent elicitation of insulin resistance that is present following resistance training counters the AMPK-driven increase in insulin sensitivity that occurs following exercise per se resulting in reduction/elimination of the PAE of exercise on insulin sensitivity following a bout of this type of exercise [93]. More research is required to further clarify the differences between resistance training and conventional endurance exercise for eliciting the PAE of exercise on insulin sensitivity and, with respect to the latter, whether specific modalities are more effective (e.g. more muscles involved vs. leg cycling, body weight supported vs. not supported, etc.).

## Frequency

To determine the frequency at which bouts must be ‘strung together’ to elicit a ‘continuous’ PAE of exercise on insulin sensitivity, the time course for dissipation of the effect must be considered. Studies that have assessed the PAE of exercise on insulin sensitivity at multiple time points provide insight. Oshida *et al*. observed elevated insulin sensitivity (EHC) in young trained athletes compared to sedentary controls at 14, but not 38 h after cessation of training [94]. King *et al*. had middle-aged moderately-trained individuals perform five consecutive days of exercise (45 min per day at ~75% ${\dot{\text{V}}\text{o}}_{2\text{peak}}$) and observed increased insulin sensitivity (OGTT) at 24 and 72, but not 90 h following the final bout [32]. In addition to age, another possible explanation for these contrasting findings is that compared to OGTT, the higher insulin stimulus during EHC might have resulted in complete suppression of hepatic glucose production thereby rendering the test incapable of detecting an increase in hepatic insulin sensitivity that might have a longer time course compared to skeletal muscle [32]. However, 72-h duration cannot be confirmed or refuted by most studies that have used EHC (the ‘gold standard’ means of assessment) to determine the PAE of exercise on insulin sensitivity because they have only involved a single measurement; for example, at ≤24 h [57,79] or 48 h [54,62]. One exception is Richards *et al*. who found unchanged insulin sensitivity in sedentary/recreationally-active individuals 72 h after the first bout of a 14-day protocol involving sprint interval training (SIT), a form of high-intensity interval training (HIIT; see below) [77]. This agrees with findings by Whyte *et al*. that increased insulin sensitivity (OGTT) following a 2-week SIT intervention was no longer present 72 h following the final bout [95]. The reason that these reports contrast those by King *et al*. might be related to volume of exercise (4–6 30-s all-out cycling sprints compared to five 45-min bouts over multiple days) [32,77,95]. In this regard, Magkos *et al*. observed a curvilinear relationship between energy expenditure during evening exercise (30–120 min on a treadmill or cycle ergometer at 60% ${\dot{\text{V}}\text{o}}_{2\max}$) and increased insulin sensitivity (HOMA) the following morning indicating that outlay ≥900 kcal was required to elicit the effect for healthy, nonobese untrained men [71]. However, this blanket statement should be interpreted with caution because in addition to energy use per se, energy use per unit time (i.e. intensity) is also important to consider (see below) [60,83; *c.f.* 61]. Finally, post bout nutrition influences the duration of the PAE of exercise on insulin sensitivity as carbohydrate ingestion has the potential to decrease the time course from 48 to 18 h in rats [40] and eliminate it completely in humans [76]. More research involving measurement of the PAE of exercise on insulin sensitivity at multiple time points is required to clarify how long the effect lasts (and, by extension, how frequently bouts should be performed) in different types of participants (e.g. healthy, but at risk for a loss of insulin sensitivity with aging vs. at risk of developing or already possessing insulin resistance vs. already possessing T2D) with different nutritional intake pre- and immediately post-bout.

## Duration

Pharmacological evaluation includes determining a drug’s minimum effective dose (MED) to inform prescription in an effective/well tolerated manner. For exercise to be considered medicine [7,8], similar determinations are required. With respect to prescribing exercise to elicit the PAE of exercise on insulin sensitivity, in addition to frequency, duration of the exercise bout is important to consider. However, like frequency, duration likely depends on intensity so establishing a single optimal frequency/duration combination is attractive in theory, but flawed in practice. Indeed, unlike prescribing pharmaceuticals, manipulating the ‘exercise dose’ is far more complicated because there are more variables to consider. Moreover, not all ‘side effects’ associated with exceeding MED in the exercise setting are deleterious. For example, while excessive volume (i.e. frequency × duration) might increase the likelihood of overuse injury and lack of program adherence, the MED required to achieve/maintain the PAE of exercise on insulin sensitivity might be less than what is necessary to establish an energy deficit to lose body fat at a sufficient rate for those who are also exercising for this purpose. This is important given the association between insulin resistance and the overweight/obese condition. However, for individuals in the MONW subgroup [96], exceeding the MED for the PAE of exercise on insulin sensitivity for this purpose would be unnecessary.

As previously mentioned, Magkos *et al*. suggest that the PAE of exercise on insulin sensitivity increased proportionally with exercise-bout energy expenditure as long as a 900-kcal threshold was surpassed, which meant that ≥60–90 min of sustained exercise was required for healthy nonobese untrained men [71]. However, even though participants were normoglycemic, the PAE of exercise on insulin sensitivity was inversely related to baseline insulin sensitivity, which raises the possibility that less might be needed for individuals with insulin resistance. Moreover, bouts were performed at a moderate intensity (60% ${\dot{\text{V}}\text{o}}_{2\text{peak}}$) in that study [71]. In this regard, Ben Ezra *et al*. found that despite equal energy expenditure (~400 kcal), treadmill walking at 70% ${\dot{\text{V}}\text{o}}_{2\max}$ for 50 min resulted in a decreased insulin response (OGTT) 15–17 h post-exercise for untrained normoglycemic women whereas walking at 40% for 87 min did not [60]. Conversely, Ortega *et al*. found that young healthy nonobese untrained men experienced improved insulin sensitivity 48 h following continuous leg cycling that required ~570 kcal regardless of whether it was performed at ~46% ${\dot{\text{V}}\text{o}}_{2\text{peak}}$ for 60 min or ~77% ${\dot{\text{V}}\text{o}}_{2\text{peak}}$ for ~35 min [83]. Moreover, despite requiring ~25% of the energy outlay of the two continuous bouts, similar improvement was derived from a bout comprising four 30-s all-out sprints (SIT) [83]. While such time efficiency of HIIT/SIT has been highly touted [97], it is important to recognise that these sprints were separated by 270 s of unloaded cycling; hence, the entire bout excluding warm-up and cool-down still required >15 min to perform [83]. Interestingly, Whyte *et al*. found that a similar SIT protocol resulted in no improvement in insulin sensitivity (OGTT) for inactive overweight/obese young men 18–22 h post-exercise whereas the same amount of work performed during a single extended sprint (i.e. ‘all-out’ cycling for ~200 s) resulted in marked improvement [80]. The authors advance this as evidence that including warm-up and cool-down, as little as 10–12 min of exercise is required to derive the PAE of exercise on insulin sensitivity [80]. More research involving measurement following exercise bouts with different duration/intensity combinations is required to clarify what is required to elicit the PAE of exercise on insulin sensitivity for different types of participants (e.g. healthy, at risk for disease or already with disease).

## Intensity

As indicated by mention in previous sections, in all likelihood, intensity is the variable that provides the foundation upon which prescription of the PAE of exercise on insulin sensitivity should be built. With respect to continuous exercise at a sustainable work rate, Braun *et al*. found that energy-matched treadmill exercise at ~50 and ~75% ${\dot{\text{V}}\text{o}}_{2\max}$ increased insulin sensitivity (insulin suppression test; IST) to a similar extent for obese women with T2D [61]. Rynders *et al*. also report similar enhancement of insulin sensitivity (OGTT) 3 h following energy-matched cycling at ~50 and ~83% ${\dot{\text{V}}\text{o}}_{2\text{peak}}$ for prediabetic individuals [82]. These findings, which suggest that energy expenditure per se is the key determinant, contrast the observation that normoglycemic untrained women achieved the PAE of exercise on insulin sensitivity (OGTT) from exercise at ~70%, but not energy-matched exercise at ~40% ${\dot{\text{V}}\text{o}}_{2\max}$ (see above) [60]. Furthermore, Newsom *et al*. observed ~35% improvement in insulin sensitivity (EHC) for sedentary obese adults ~19 h after participants expended 350 kcal at ~50% ${\dot{\text{V}}\text{o}}_{2\max}$ whereas similar expenditure at ~65% elicited a nonsignificant ~20% increase [79]. On the surface, equal if not greater enhancement at energy-matched lower-intensity exercise is counterintuitive being that, presumably, the PAE of exercise on insulin sensitivity is at least in part determined by muscle-glycogen depletion [41]. However, Newsom *et al*. also report a correlation between the PAE of exercise on insulin sensitivity and the change in fatty acid rate of disappearance from plasma, which was reduced following the ~50%, but not ~65% protocol [79]. Given the link between skeletal-muscle fatty acid uptake and the obesity-related decline in insulin action [98], this raises the possibility that exercise of lower intensity can also play a role in establishing the PAE of exercise on insulin sensitivity via its effect on lipid metabolism even though it does not substantially influence muscle-glycogen stores [79].

More information regarding intensity and the PAE of exercise on insulin sensitivity comes from research that has ‘stretched’ the intensity extreme by having subjects perform intermittent exercise involving unsustainable work rates (HIIT) or exercise with all-out effort (SIT) [99]. As previously mentioned, Ortega *et al*. found similar enhancement of insulin sensitivity (IVGTT) for young healthy nonobese untrained men 48 h following exercise regardless of whether it involved four 30-second cycling sprints or continuous cycling at moderate or high intensity that required four times the energy expenditure of the sprint bouts [83]. While this finding suggests intensity is important to consider, the fact that PAE of exercise on insulin sensitivity induced by the energy-matched continuous bouts in that study did not differ indicates that energy expenditure per se is a key determinant at least during sustainable exercise. Conversely, Brestoff *et al*. found that SIT did not elicit a similar effect as continuous cycling in recreationally-active individuals who experienced no change in insulin sensitivity (OGTT) 12–16 h after completing five 30-s sprints separated by 240–300 s whereas a 70–100% increase was present following 45 min of cycling at ~75% ${\dot{\text{V}}\text{o}}_{2\max}$ [72]. Importantly, energy expenditure was not matched for the two conditions; hence, the lesser outlay during SIT might have played a role [72; *c.f*., 83]. Using the same methodology, Metcalfe *et al*. found that ‘reduced-exertion’ SIT (two 20-s sprints separated by 200 s) also did not induce PAE on insulin sensitivity; however, in that study, the 45-min bout also elicited no effect [85]. Baseline insulin sensitivity and post-exercise feeding are two factors that might explain this unexpected finding [85]. Finally, while Richards *et al*. observed increased insulin sensitivity following 14 days of SIT for sedentary/recreationally-active individuals, there was no acute effect after the initial bout of the protocol (four 30-s sprints separated by 240 s) [77]. However, as previously mentioned, in that study, the PAE of exercise on insulin sensitivity was only assessed (EHC) 72 h after completion of the initial bout [77], which means that the degree to which the effect might have been present earlier cannot be determined. However, in this regard, Whyte *et al*. found no increase in insulin sensitivity (OGTT) 24 h following SIT (four 30-s sprints separated by 270 s) although as previously mentioned, insulin sensitivity was increased when recovery intervals were removed from an all-out work-matched bout [80]. More research is required to determine whether it is energy use per se or energy use per unit time (i.e. intensity) which is the primary factor that drives the PAE of exercise on insulin sensitivity and whether low-intensity sustained exercise that maximizes fat use, high-intensity sustained exercise that maximizes energy use (e.g. at the ‘critical power/velocity’) or very-high-intensity unsustainable intermittent exercise that provides for maximal mitochondrial stimulation and glycogen depletion provides for the optimal PAE of exercise on insulin sensitivity.

### Conclusion

Exercise is medicine implies that exercise can serve as a first-line defense for prevention/treatment of disease with pharmaceuticals representing a surrogate for exercise only when necessary. Regular exercise results in chronic adaptations that help serve this purpose; however, while recognized for many years, there is growing appreciation for beneficial residual effects of each individual exercise bout on myriad aspects of function [100,101]. With respect to insulin sensitivity, the PAE of exercise appears to be the predominant effect induced by training even for well-trained individuals with high insulin sensitivity [37]. Furthermore, untrained individuals [47] and individuals with lower insulin sensitivity [57,71] benefit more from the PAE of exercise whereas the chronic-exercise effect on insulin sensitivity for individuals with ‘metabolic syndrome’ might depend on weight loss that accompanies exercise as opposed to exercise per se [102,103]. This means that for at-risk individuals for whom it is not necessary or feasible to lose weight (MONW, for example), the PAE of exercise on insulin sensitivity might be the only option available. Collectively, these observations suggest that an intervention designed to combat the demise in insulin sensitivity that begins the pathological journey to cardiometabolic diseases including T2D should be prescribed with precision to elicit a PAE of exercise on insulin sensitivity that can serve as a surrogate for pharmaceutical intervention or, in cases where such intervention is necessary, an adjunct to it [104]. While prescribing exercise is far more complicated than prescribing pharmaceuticals due to the many variables that can be manipulated (e.g. mode, volume, frequency, duration and, perhaps most importantly, intensity), beginning with the seminal work of the Holloszy group in the 1980s, there have been a number of studies that have provided information regarding the PAE of exercise on insulin sensitivity that are important to consider for determining the specifics of prescribing exercise to elicit/maintain this effect. Table 1 provides a chronically-ordered listing of some of the major ones involving human subjects. However, more standardised research with gold-standard testing (e.g. EHC, as opposed to assessments used in many of the studies included in this review; e.g. OGTT, ITT, IST and HOMA) would be helpful to further determine dose-response specifics (e.g. session frequency and duration, importance of energy use per se vs. intensity of effort and degree to which lower- and higher-intensity efforts elicit the PAE of exercise on insulin sensitivity via different mechanisms) for individuals with varying degrees of insulin sensitivity/insulin resistance or T2D. Differentiation between the minimal effective and optimal dose is also important to clarify because many of the individuals that can benefit most from the PAE of exercise on insulin sensitivity might be unable to achieve/sustain the latter. Finally, the influence of post-exercise feeding requires further investigation because the PAE of exercise on insulin sensitivity can be eliminated via carbohydrate replenishment despite negative energy balance or maintained despite energy replenishment with carbohydrate restriction [76].

## Acknowledgements

**Conflicts of interest**

There are no conflicts of interest.

## Supplementary Material


